# Supplementary material for: Evaluation of expression variations in virulence-related genes of Leishmania major after several culture passages compared with Phlebotomus papatasi isolated promastigotes
Source: PLoS One. 2023 Apr 13;18(4):e0284240. doi: 10.1371/journal.pone.0284240 (PMC10101501; doi:10.1371/journal.pone.0284240)
Supplement: Attachment — Submitted filename: Response to the comments.docx [file pone.0284240.s001.docx]

PONE-D-22-35287

Evaluation of Expression variations in virulence-related genes of Leishmania major after several culture passages compared with Phlebotomus papatasi isolated promastigotes

PLOS ONE

Dear editor and reviewers

The authors thank for spending time on this MS and giving very valuable comments; here, we have prepared the revised version and hope to find it appropriate.

**Editors comments:**

1. Please ensure that your manuscript meets PLOS ONE's style requirements, including those for file naming.
2. Thank you for including the following ethics statement on the submission details page:
   'The manuscript was ethically approved by the Ethics Committee of Isfahan University of Medical Sciences, Isfahan, Iran (Code No: 398634).' Please specify if the approval includes both animal and human research in your study. In addition, please report the method of animal euthanasia in the Methods section.

Response: the euthanasia method was added to the methods section.

3. Please provide additional details regarding participant consent. In the ethics statement in the Methods and online submission information, please ensure that you have specified (1) whether consent was informed and (2) what type you obtained (for instance, written or verbal, and if verbal, how it was documented and witnessed). If your study included minors, state whether you obtained consent from parents or guardians. If the need for consent was waived by the ethics committee, please include this information.

Response: no animal studies were performed. Regarding human samples, we only indirectly gathered those samples that had already been taken from patients by the Institute Pasteur of Iran; hence, we did not directly involve in the sampling procedure.

4. In your Data Availability statement, you have not specified where the minimal data set underlying the results described in your manuscript can be found.
5. PLOS requires an ORCID iD for the corresponding author in Editorial Manager on papers submitted after December 6th, 2016.

Response: the ORCID ID was matched for the corresponding author.

**Reviewers’ comments:**

**Reviewer #1:** The manuscript evaluates the expression variations in virulence-related genes of L. major after several culture passages compared with wild parasites. The title sound novel and would represent valuable information on virulence-related genes that can be finally translated into the clinical settings.
The utilized methods have been appropriately applied towards the manuscript goal. There is only some typographical and stylistic errors in English writing in the manuscript, mostly in introduction and discussion parts, which should be obviated.
In total, the manuscript has been carefully devised and performed, and will be of great interest to the readers of PLoS One journal.

Response: with thanks; some sections were improved as highlighted.

**Reviewer #2:** This manuscript presents an interesting concept and is well designed. There are some comments that must be addressed:
-Please write the abstract in a structural form. See the journal guidelines.
-Line 41-42: Please write the full form of gp63, HSP70, LACK, SHERP, CPB in the abstract first, and then write its abbreviation.
-Line 51: Please correct the phrase "Out results showed a significant increased gene..."
-Line 52-54: Please state any significant differences in the abstract text statistically and write p-value.
- According to the sentence of line 99. Explain the reason for choosing the Stationary phase compared whit the logarithmic phase.
-Please mention the reason for choosing seven genes among different virulence factors in the introduction section.
-In line 125, please write Ph. papatasi in italic form and consider in other parts of the text.
-In line 182: write the type of Real Time PCR system and Master Mix in detail.
- In line 185, it refers to the (Table), while there is no table in the text of the manuscript. Please check.
-Please mention the limitations and recommendations in the last paragraph of the discussion.

Response: with thanks for your above constructive comments; all of the comments were obviated in the manuscript text, as highlighted in yellow.
